# Prerequisites for ethical leadership: insights from health and social care leaders

**DOI:** 10.1108/LHS-12-2025-0201

**Published:** 2026-06-30

**Authors:** Anniina Seere, Riitta Suhonen, Johanna Wiisak

**Affiliations:** Department of Nursing Science, University of Turku, Turku, Finland; Department of Nursing Science, University of Turku, Turku, Finland, and Turku University Hospital, Wellbeing Services County of Southwest, Finland, Finland; Marie Curie Fellow, Lero – The Irish Software Research Centre, J E Cairnes School of Business and Economics, University of Galway, Galway, Ireland, and Department of Nursing Science, University of Turku, Turku, Finland

**Keywords:** Ethical leadership, Ethical agency, Health and social care, Prerequisites for ethical leadership, Reflexive thematic analysis, Leadership development

## Abstract

**Purpose:**

This study aims to explore frontline and middle-management leaders’ experiences of ethical leadership in health and social care and examine the prerequisites associated with its enactment in everyday work.

**Design/methodology/approach:**

Semi-structured interviews were conducted with 16 health and social care leaders and analysed using Braun and Clarke’s reflexive thematic analysis. The study adhered to the Standards for Reporting Qualitative Research and the Consolidated Criteria for Reporting Qualitative Research.

**Findings:**

Five interrelated themes described the prerequisites for ethical leadership: its fundamental value-based nature; leaders’ internal ethical capability; human interaction and trust; organisational structures and culture; and ethical agency within hierarchical systems. Ethical leadership was described as a central aspect of managerial work, grounded in client-centredness and professional integrity, yet its enactment was perceived to be constrained in contexts characterised by unclear strategic direction, hierarchical pressures and competing organisational demands.

**Practical implications:**

Strengthening ethical leadership may require multi-level support addressing individual, relational and organisational conditions. Organisations can foster these prerequisites through coherent strategic direction, transparent decision-making processes and systemic investment in relational practice. Supporting these conditions may help reduce moral distress, improve staff retention and enhance patient safety by enabling ethical leadership to be consistently enacted in practice.

**Originality/value:**

This study provides new empirical insight into the prerequisites associated with ethical leadership in health and social care. By reframing ethical leadership from an individual attribute to a systemic capability, the study highlights the individual, relational and organisational conditions associated with sustaining ethical practice in complex health and social care environments.

## Introduction

Leadership in health and social care is inherently ethical, as leaders’ decisions are closely linked to care quality, staff well-being, service outcomes and organisational integrity (Gallagher and Tschudin, 2010; Northouse, 2021). Ethical leadership is increasingly recognised as an important factor associated with equitable, value-based and high-quality care, as well as contributing to organisational sustainability, workforce retention and patient safety (Lotfi *et al.*, 2018; Singh and Vashist, 2024).

While ethical leadership is widely recognised as important, its enactment is not always straightforward and may be constrained by contextual conditions (Ayan and Baykal, 2023; Storaker *et al.*, 2022). These conditions can therefore be understood as prerequisites for ethical leadership. Their development represents not only an ethical concern but also a strategic organisational consideration related to workforce sustainability and system resilience (OECD/European Commission, 2024).

In practice, supporting these prerequisites can be challenging. Leaders must balance staff needs, organisational expectations and resource constraints while remaining accountable for well-being and regulatory compliance (Ayan and Baykal, 2023; Barkhordari-Sharifabad *et al.*, 2017). Structural limitations and unsupportive organisational cultures may further restrict leaders’ capacity (Storaker *et al.*, 2022), whereas barriers such as inadequate support, resource scarcity and ineffective communication constitute persistent obstacles to ethical leadership (El-Gazar and Zoromba, 2021; Morin and Talbot, 2023). Systematic efforts to strengthen these prerequisites remain limited, reflecting an incomplete understanding of how they are experienced and enacted in everyday leadership practice (Singh and Vashist, 2024).

Ethical leadership is commonly defined as normatively appropriate conduct expressed through personal behaviour and relationships and reinforced through communication (Brown and Treviño, 2006), while contemporary perspectives increasingly emphasise its interactional and context-dependent nature grounded in fairness, responsibility and shared values (Eisenbeiss, 2012; Northouse, 2021). These perspectives also highlight the role of organisational conditions in shaping how ethical leadership is enacted in practice (Morin and Talbot, 2023; Storaker *et al.*, 2022). Prerequisites for ethical leadership can therefore be understood as combinations of individual competencies (Barkhordari-Sharifabad *et al.*, 2017; Makaroff *et al.*, 2014) and organisational conditions (Scharf and Berntson, 2025) and together these prerequisites may constitute a broader foundation for ethical leadership.

Existing research has predominantly examined the outcomes of ethical leadership using quantitative approaches (Singh and Vashist, 2024), while qualitative studies have focused on identifying barriers in specific contexts (Barkhordari-Sharifabad *et al.*, 2017). However, the prerequisites for ethical leadership – the conditions that enable its enactment in practice – remain underexplored. A recent literature review identified five categories of such prerequisites in health and social care: leader-centric competences, ethical organisational culture, leadership support, ethical guidelines and resource sufficiency (Seere *et al.*, 2026). Yet, prerequisites have largely remained a secondary focus of research, particularly from the perspectives of leaders themselves.

This study responds to this gap. Drawing on leaders’ lived experiences, it extends ethical leadership research by shifting the focus from individual leader characteristics towards the organisational and relational conditions shaping ethical leadership practice. The aim of this study was to explore frontline and middle-management leaders’ experiences of ethical leadership in health and social care and to examine the prerequisites associated with its enactment in everyday work.

## Methods

### Design

A qualitative design was used to explore the complex and context-dependent nature of ethical leadership in health and social care. Reflexive thematic analysis (RTA) was chosen for its flexibility in capturing both semantic and latent meanings and for acknowledging the researcher’s interpretive role (Braun and Clarke, 2006, 2021). This approach enabled the examination of leaders’ lived experiences in relation to the conditions that shape ethical leadership. The study and its reporting followed the Standards for Reporting Qualitative Research (SRQR) to ensure overall methodological rigour and the Consolidated Criteria for Reporting Qualitative Research (COREQ) to support transparency in interview-based research (O'Brien *et al.*, 2014; Tong *et al.*, 2007) (see Supplementary material File 1).

Guided by critical realism, the study examined both participants’ experiences and the organisational conditions, including structural elements that shape them. This perspective distinguishes between reported experiences, the mechanisms through which they arise, and the broader structural conditions, such as governance and institutional culture, that influence these mechanisms (Bhaskar, 2008; Fletcher, 2017). Ethical leadership was thus conceptualised not only as an individual practice but as a phenomenon shaped by organisational conditions and their underlying mechanisms.

### Study setting and recruitment

Data were collected between January and March 2025 in public health and social care organisations. Using purposive sampling, 16 frontline and middle-management leaders were recruited from well-being services counties across four of Finland’s five national collaboration areas to capture geographical and organisational variation (Ministry of Social Affairs and Health, 2024). The target group comprised leaders with current supervisory roles, identified with the assistance of local contact persons (e.g. chief nursing officers). Sample adequacy was considered with reference to the concept of information power (Malterud *et al.*, 2016), which guided the assessment of the richness and relevancy of the data in relation to the study aim. Consideration was given to the focused study aim, the specificity of the sample, the applied theoretical framework, the quality of dialogue and the analytic strategy. Ongoing reflexive assessment supported the judgement that sufficient information power had been reached, with later interviews confirming and deepening the emerging themes without introducing substantially new perspectives.

### Data collection

Before interviews, participants received information about the study purpose, procedures and confidentiality and provided informed consent. Data were collected through face-to-face (*n* = 6) or secure online interviews (*n* = 10), depending on participant preference. All interviews were conducted privately and without interruptions, lasting 45–75 min (*M* = 55).

The interviews were conducted by a trained qualitative researcher with professional experience in health and social care. This background informed the interview process and reflexive awareness on its potential influence was maintained throughout data collection. A semi-structured interview guide, developed from relevant literature, research aims and researcher expertise, was refined based on feedback from another member of the research team. Preliminary test interviews were conducted with health and social care leaders who did not participate in the study to ensure clarity and relevance; no revisions were required. The first four interviews were additionally monitored closely for consistency with the guide, further confirming its adequacy. The guide covered six topic areas, including perceptions and prerequisites of ethical leadership (Table 1). Data collection continued until sufficient information power was achieved (Malterud *et al.*, 2016). All interviews were audio-recorded and transcribed *verbatim* immediately after completion.

**Table 1.** Semi-structured interview guide themes and components

Theme	Focus area	Key components
Background and context	Understanding respondent’s work environment	Work background, education, field, typical workday, scientific definition of ethical leadership
General perceptions	Overall understanding of ethical leadership	Personal definitions, organisational emphasis on ethical leadership
Work experiences	Personal encounters with ethical leadership	Specific examples of important ethical leadership situations, instances of failed ethical leadership
Prerequisites and requirements	Conditions needed for ethical leadership	Supporting organisational factors, necessary competencies, guidelines and principles, required resources and support
Challenges and obstacles	Barriers to ethical leadership	Major obstacles encountered in practice
Development and support	Improvement opportunities	Better practices, promoting ethical culture, supporting ethical leadership
Final reflection	Broader considerations	Additional thoughts, unaddressed important aspects

**Source(s):** Authors’ own work

### Ethical statement

The study adhered to international and national ethical guidelines (All European Academies, 2017; Committee on Publication Ethics, 2023). Organisational research approvals were obtained before data collection. In accordance with national research ethics regulations, a formal ethics review was not required because participants were professionals and no patient data or sensitive personal information were collected. All participants received written information about the study, confidentiality was assured and they were reminded of their right to withdraw at any point without consequences. Written informed consent was obtained from all participants.

Data were pseudonymised using numerical codes and handled in compliance with the General Data Protection Regulation (European Union, 2016). Data were stored securely and are not publicly available. Reflexivity was maintained throughout data collection and analysis through memo writing and journaling. The research team treated researchers’ professional backgrounds as analytical resources within the interpretive process (Fletcher, 2017).

Trustworthiness was supported following established qualitative criteria (credibility, transferability, dependability and confirmability) through transparent documentation, purposive sampling and thick description (Lincoln and Guba, 1985). RTA was conducted systematically, guided by Braun and Clarke’s methodological principles and their 20-point checklist (Braun and Clarke, 2006, 2019, 2021) (see Supplementary material File 2).

### Data analysis

Following transcription, all interviews were checked for accuracy and imported into NVivo 14 for coding. Data were analysed using RTA as outlined by Braun and Clarke (2006, 2019, 2021), informed by a critical realist framework with constructionist orientation, focusing on both explicit meanings and underlying ideas. The analysis followed Braun and Clarke’s six phases (Table 2) and was conducted iteratively by moving between phases to deepen interpretation.

**Table 2.** RTA process (Braun and Clarke, 2006, 2019) adapted for this study

Phase	Description (including the specific procedures for this study)
Familiarisation	Each interview was listened to, transcribed verbatim and checked for accuracy. Transcripts were read repeatedly February–May 2025. A reflexive diary captured initial impressions, positional reflections and early analytic insights
Initial coding	Inductive, interpretative coding was conducted in NVivo 14. The first four interviews were double-coded for consistency. Codes captured both semantic and latent meanings, expanding from 67 to 174, covering 4,117 excerpts
Theme development	Codes were grouped into progressively broader meaning patterns (174 → 47 clusters → 24 groups → 9 subthemes). Analytical interpretation guided the move from description toward conceptual patterning
Reviewing and refining	Potential subthemes were critically reviewed against the full dataset for coherence and distinction. Through iterative comparison, six preliminary themes were refined and restructured to capture the data’s central meanings
Naming and final analysis	The six themes were further refined into five final themes. Each was named according to its central organising concept, with subthemes representing internal variation
Reporting	The final analytic narrative integrated all five themes, supported by pseudonymised quotations and presented as a coherent and theoretically grounded account of the prerequisites for ethical leadership

**Source(s):** Authors’ own work

Coding and theme development were conducted by one member of the research team, which is consistent with the principles of RTA, where the analytic process is understood as inherently interpretive rather than requiring inter-rater agreement (Braun and Clarke, 2019, 2021). Two other team members reviewed the coding structure, theme development and manuscript drafts, with the opportunity to raise concerns or suggest revisions throughout the process. The first author's professional background in health and social care was recognised as a potential source of interpretive influence and was systematically addressed through reflexive journaling during the analytic process.

All 16 interviews were included in the final analysis. In total, 174 codes were generated from 4,117 references and grouped into initial meaning patterns, which were iteratively reviewed and refined into five final themes addressing the research question.

The interpretation drew on Fryer’s (2022) critical realist perspective, viewing ethical leadership as shaped by both individual and organisational factors. This was reflected in the thematic structure, which encompasses leaders’ personal experiences and capacities as well as the conditions in which ethical leadership is enacted. The analytic process is further demonstrated in Table 3 with example codes and illustrative quotes and in Supplementary material File 3 with more detailed coding examples and illustrative quotes. Themes were developed inductively, focusing on the meaning and relevance of codes rather than their frequency to ensure a coherent and layered interpretation (Braun and Clarke, 2006, 2021).

**Table 3.** Example quotes, subthemes and themes

Illustrative quote	Subthemes (examples)	Theme
“Always a bit like, from whose perspective is it ethical and whose values are these ” (P13)	Complexity and boundary work in ethical leadership	Theme 1. The fundamental nature of ethical leadership
“It requires self-examination and self-awareness… it doesn’t just come as a birthright ” (P16)	Developing ethical leadership competence	Theme 2. The leader’s internal ethical capability
“You can’t be ethical alone in an unethical group ” (P14)	Community, peer support and psychological safety	Theme 3. Human interaction and trust
“We’re in a severe financial squeeze… that’s really steering decisions ” (P12)	Economic and political constraints shaping leadership	Theme 4. Organisational structures and culture
“There’s so much responsibility, so there has to be power, too ” (P5)	Hierarchical and communicative constraints	Theme 5. Ethical agency in hierarchical systems

**Source(s):** Authors’ own work

Rather than operating as discrete categories, these themes reflect how ethical leadership is understood across multiple contexts, highlighting its embedded and context-dependent nature. From a critical realist perspective, the themes were examined across three complementary analytical layers: leaders’ lived experiences, the situations in which ethical leadership is enacted, and the organisational conditions, including structural elements, that may influence ethical practice. Finally, the themes were examined in relation to their underlying connections and patterns and were analytically synthesised to identify broader dimensions that capture how prerequisites for ethical leadership are structured and enacted across different contexts.

## Results

### Characteristics of participants

The study included 16 health and social care leaders representing frontline and middle-management positions across diverse organisational contexts. The participants were predominantly women, highly educated, with leadership experience ranging from less than 5 years to more than 20 years. Variation emerged in leadership levels, sectors and years of experience, providing multiple perspectives. Detailed characteristics are provided in Table 4.

**Table 4.** Sociodemographic characteristics of participants (*n* = 16)

Characteristics	Participants
*Leadership experience (years)*	
1–5	2
5–10	2
10–15	3
15–20	5
Over 20	4
*Gender*	
Male	1
Female	15
*Education level*	
Bachelor’s degree	2
Master’s degree	13
Doctoral degree	1
(of which with JET*)	6
*Leadership level*	
Frontline	7
Middle management (e.g. chief officer)	9

Note(s):

*JET = specialist qualification in management

**Source(s):** Authors’ own work

### Overview of themes

**Figure 1.**
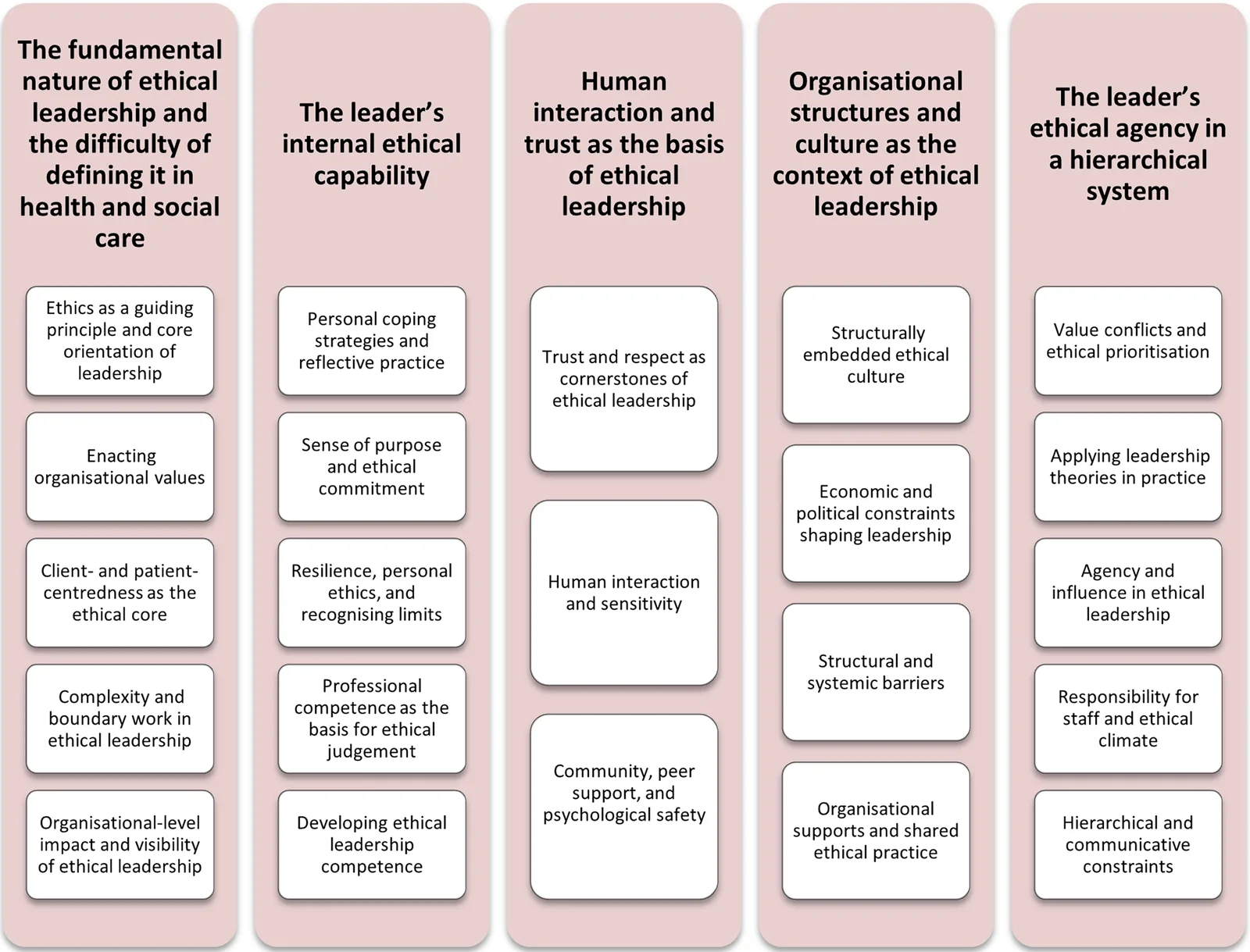
Overview of themes and subthemes **Source:** Authors’ own work

The analysis identified five interrelated themes describing the prerequisites for ethical leadership in health and social care: the fundamental nature of ethical leadership, the leader’s internal ethical capability, human interaction and trust, organisational structures and culture and ethical agency in hierarchical systems. The final thematic structure, including the themes and subthemes, is presented in Figure 1. Each theme is presented in the following subsections with analytic interpretation and pseudonymised participant quotes. The themes were interpreted across individual, interactional and organisational levels, highlighting ethical leadership as a multi-level phenomenon shaped by personal, relational and structural conditions.

#### Theme 1: the fundamental nature of ethical leadership and the difficulty of defining it in health and social care.

Ethical leadership was described as the moral foundation of all leadership practice, an inseparable aspect of good leadership that gives direction and legitimacy. This positioned ethical leadership not as a separate leadership style, but as an underlying aspect of all leadership activities. The participants highlighted the ethical and legal obligations of the sector while acknowledging the difficulty of consistently defining ethics in everyday decision-making.

### Ethics as a guiding principle and core orientation of leadership

The leaders described ethics as the foundational principle of leadership, not an additional component but the core that directs decisions and everyday practices. Ethical leadership was presented as an inherent expectation in health and social care. At the same time, this taken-for-granted nature made ethics less visible and more difficult to articulate in practice. The participants emphasised that shared values, continuous reflection and open discussion are required for ethical leadership to remain a lived practice and not treated as rule-based compliance: “It’s the same as good leadership itself” (Participant 10).

### Enacting organisational values

The leaders described justice, legality and equality as the basis of ethical leadership. However, they noted that organisational values were not always made visible or translated into daily practice, which created a perceived gap between formally stated values and their enactment in everyday work: “I don’t even remember what our organisation’s values are” (Participant 1).

### Client- and patient-centredness as an ethical core

The leaders viewed ethical leadership as fundamentally client- and patient-centred, emphasising dignity, autonomy and fairness: “If the client wants something, it has to be provided, they have the right to self-determination” (Participant 1). Ethical action meant keeping the client’s best interest central even under organisational pressure. This also brought forward situations where patient-centred values had to be balanced with organisational constraints.

### Complexity and boundary work in ethical leadership

The participants described ethical leadership as complex and context-dependent, involving continuous negotiation between competing values and expectations: “Always a bit like, from whose perspective is it ethical and whose values are these” (Participant 13). Ethical decision-making was described as requiring a tolerance of uncertainty, reflective sensitivity and courage to address difficult issues. This positioned ethical leadership as a context-dependent and continuously negotiated process.

### Organisational-level impact and visibility of ethical leadership

Ethical leadership was described as an organisation-wide phenomenon embedded in structures, culture and daily practices, rather than solely in individual leaders’ actions. The leaders emphasised that ethical leadership required shared principles and coherence across organisational levels. Its effects were described to extend beyond staff experiences to the quality of care and client outcomes: “That uncertainty spreads and is felt in the staff, so of course it affects the clients” (Participant 6). This highlighted how ethical leadership was understood as relational and systemic, influencing both workplace well-being and care practices.

#### Theme 2: the leader’s internal ethical capability.

The leaders described ethical capability as an inner resource developed through self-awareness, emotional resilience and professional competence. Sustaining ethical leadership appeared to involve a balance of personal reflection, value-based decision-making and continual learning in demanding organisational contexts. This positioned ethical capability as something actively maintained rather than a fixed individual attribute.

### Personal coping strategies and reflective practice

The participants described conscious strategies to manage ethical strain when organisational demands conflicted with their values. Recognising emotions and practising self-compassion were viewed as important for sustaining motivation: “I’ve learned to be merciful to myself” (Participant 8). The leaders also noted the need to actively protect value-based commitment. These strategies appeared to support the maintenance of ethical orientation under pressure.

### Sense of purpose and ethical commitment

The leaders described meaning and necessity as key resources for sustaining ethical commitment. Returning to core values helped clarify priorities, while opportunities to influence decisions and understand their rationale were associated with greater sense of calmness and motivation in ethically challenging contexts: “Sometimes you have to go back to that core task, to remember why you’re here” (Participant 15). This highlighted the role of purpose in stabilising ethical decision-making in uncertain situations.

### Resilience, personal ethics and recognising limits

Ethical leadership was described as grounded in personal values and self-reflection rather than rules. The leaders expressed a fear of becoming cynical, and they emphasised recognising personal limits as responsible boundary-setting: “The greatest danger for a supervisor is becoming really cynical” (Participant 4). Recognising limits was interpreted as part of maintaining ethical responsibility rather than as a limitation of leadership.

### Professional competence as the basis for ethical judgement

Ethical leadership was viewed as requiring sufficient education and domain-specific expertise, not only moral intent. The leaders described formal qualifications and updated knowledge as essential for making responsible decisions: “A supervisor background education is sufficient. Now we’ve set that you need that higher degree” (Participant 12). Professional competence was thus viewed as a key condition associated with ethically grounded leadership.

### Developing ethical leadership competence

Ethical leadership was described as a lifelong learning process, shaped through professional knowledge, experience and reflection. Growth increased both competence and awareness of ethical complexity and personal incompleteness: “Every course, every lesson adds, but also increases, understanding of how much there is to learn” (Participant 14). This illustrated how ethical capability was understood as continuously evolving rather than fully attainable.

#### Theme 3: human interaction and trust as the basis of ethical leadership.

The leaders described ethical leadership as inherently relational, rooted in communication, mutual trust and respect. Human interaction was not described as an accessory but as a central condition associated with ethical decision-making, value alignment and psychological safety in everyday leadership. In this way, ethical leadership was interpreted as emerging through relational processes rather than residing solely in individual leaders.

### Trust and respect as cornerstones of ethical leadership

Trust was viewed as a reciprocal basis for open dialogue, psychological safety and value-based decision-making: “There has to be that kind of mutual trust so you can lead ethically, with cooperation and fairness” (Participant 13). Respect was expressed through listening, fairness, dignity and recognition of professional expertise, supported by feedback and autonomy. Trust and respect were described as mutually reinforcing relational conditions linked to consistent ethical action.

### Human interaction and sensitivity

Leaders considered humane, emotionally attuned interaction essential for ethical leadership. Listening was described as a core skill that builds trust and understanding: “Listening and hearing are certainly one big part […] something we could actually do more of” (Participant 2). Time pressures were described as limiting leaders’ ability to be genuinely present, requiring a constant balance between efficiency, empathy and sensitivity to individual situations. This highlighted the practical tension between relational leadership ideals and organisational demands.

### Community, peer support and psychological safety

Ethical leadership was described as a collective process requiring shared values, open dialogue and psychological safety. Peer support was linked to sustaining motivation and ethical clarity: “It really is a prerequisite, that support, for being able to truly keep going as a leader” (Participant 16). The leaders emphasised that ethical leadership cannot be enacted alone but requires collegial trust, space for reflection and a supportive culture. This positioned ethical leadership as relationally sustained rather than individually enacted.

#### Theme 4: organisational structures and culture as the context of ethical leadership.

Leaders described organisational structures and culture as contextual conditions associated with the possibilities for ethical leadership. These conditions were described as shaping the extent to which ethically grounded managerial practice could be enacted in daily operations. This theme situated ethical leadership within the wider organisational environment rather than within individual leaders alone.

### Structurally embedded ethical culture

Ethical leadership was described as a collective structural responsibility requiring visible integration into strategies, guidelines and everyday decision-making. Ethics were understood as capable of operating silently and implicitly, while still guiding practice: “Maybe it’s not spoken about out loud, but it’s there in the background” (Participant 11). Clear principles and shared rules were seen to support fair and transparent action, though ambiguity in roles and responsibilities could still create tension. At the same time, the leaders also indicated that ethical culture did not become visible automatically, but appeared to require deliberate reinforcement in everyday organisational practices.

### Structural and systemic barriers

The participants described how hierarchical distance, top-down decision-making and unclear guidance limited their ethical agency. Rapid organisational changes and decisions made without consultation were thought to cause tension and uncertainty: “We are like marionette dolls, constantly receiving orders from above, but no one ever asks us” (Participant 1). These structures were described as being linked to reduced trust and hindering consistent ethical leadership and as well as increasing leaders’ sense of distance from decision-making and reducing their opportunities to act in line with shared values.

### Economic and political constraints shaping leadership

The leaders described financial pressures and political steering as major forces limiting ethical decision-making and prioritising efficiency over values: “Right now, we’re in quite severe financial trouble, so that’s really the main thing driving decisions” (Participant 12). Such conditions were described as limiting opportunities for ethical dialogue and as being associated with reduced motivation and trust, with leaders expressing concern about potential implications for care quality. The leaders also noted differing perspectives on how to sustain ethical culture when organisational priorities conflicted, illustrating tensions between ethical leadership and external demands related to economy and governance.

### Organisational supports and shared ethical practice

Leaders described supportive organisational structures, expert resources and collegial networks as essential for ethical leadership: “We have colleagues, our own supervisor, support services, all kinds of expertise available” (Participant 10). Adequate time, clear communication and shared values were associated with more consistent and transparent decision-making, while defined roles and guidelines supported coherence across the organisation. Supportive structures were thus interpreted as contributing to the sustainability of ethical leadership and reducing its reliance on individual effort alone.

#### Theme 5: the leader’s ethical agency in a hierarchical system.

The leaders described ethical agency as the ability to make and enact value-based decisions within organisational hierarchies. It was interpreted as the link between ethical principles and real-world leadership practice, shaped through the interaction of personal commitment and organisational conditions. Ethical agency was thus described as the practical realisation of ethical leadership within specific organisational contexts.

### Value conflicts and ethical prioritisation

Ethical leadership was understood to require making value-based decisions under pressure, where recognising values without acting on them was seen as insufficient. Fairness was experienced subjectively and could not be fully guaranteed, even with careful consideration: “It’s always such a personal thing how each employee experiences whether they have been treated fairly” (Participant 12). Leaders described navigating ethical principles alongside organisational and financial constraints, noting that sustained value conflict was associated with challenges in maintaining ethical leadership. This highlighted the continuous balancing required between competing expectations and values in everyday leadership.

### Applying leadership theories in practice

Ethical leadership was described as context-driven rather than theory-led, with everyday decisions relying on situational judgement instead of formal frameworks: “Not once during the day does any leadership theory actually cross my mind” (Participant 10). Unpredictable, time-pressured work was viewed as requiring flexibility, ethical sensitivity and value-based decisions rather than predefined models. Leadership practice was thus interpreted as grounded in experience and situational interpretation rather than formal theoretical application.

### Agency and influence in ethical leadership

Leaders emphasised that ethical leadership required genuine agency, not only responsibility but also the ability to influence how decisions were made and enacted. A lack of corresponding authority was seen to undermine ethical integrity: “There’s all this responsibility but not the same amount of power” (Participant 5). Having real influence and being included in decision-making processes was described as important for maintaining ethical clarity and consistency in ethical leadership. The balance between responsibility and authority was therefore interpreted as a key condition associated with ethical leadership.

### Responsibility for staff and ethical climate

Ethical leadership included responsibility for staff well-being and the moral climate of the workplace. Leaders described this as part of their ethical agency, requiring attention to employees’ coping and resilience: “You also have to look at how the staff are doing, so they don’t end up with compassion fatigue” (Participant 11). Ethical decisions were seen to involve considering how everyday choices affected well-being and fairness. This extended ethical agency beyond decision-making to include responsibility for the consequences of those decisions.

### Hierarchical and communicative constraints

Organisational hierarchy and one-way communication were viewed as restricting leaders’ ethical agency, especially when responsibility exceeded decision-making authority. The participants described how top-down decisions excluded leaders from dialogue and felt disconnected from practice: “The more layers there are, the more it stalls […]” (Participant 15). Limited reciprocal communication was linked to reduced trust and perceived influence, while transparent two-way dialogue and participatory structures were considered essential. These conditions were described as limiting leaders’ opportunities to enact ethical leadership in practice.

### Synthesis of findings

**Figure 2.**
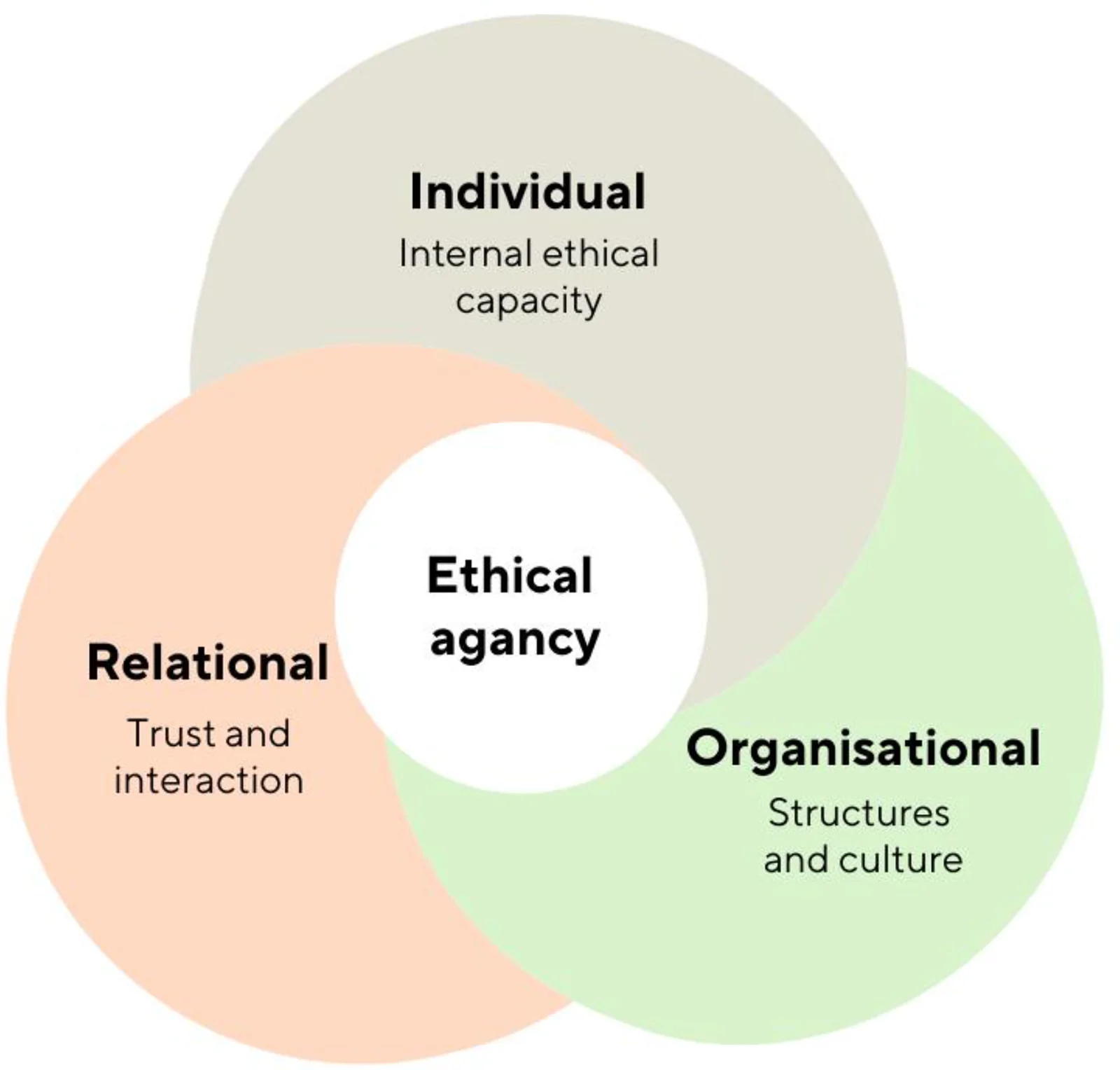
Analytical synthesis of the prerequisites for ethical leadership in health and social care **Source:** Authors’ own work

The findings suggest that ethical leadership in health and social care cannot be fully understood through individual leader attributes alone, but is also shaped by broader organisational and relational conditions. Together, the five themes illustrate three interrelated levels at which individual capability (Theme 2), relational conditions (Theme 3) and organisational culture and structures (Theme 4) function as interconnected prerequisite dimensions, whereas Theme 1 provides the conceptual foundation and Theme 5 reflects the ethical agency through which these conditions are negotiated and enacted in practice (Figure 2).

Individual ethical capability appeared insufficient in the absence of supportive relational and structural conditions. Relational trust appeared to connect individual and organisational levels, while ethical agency was shaped by the balance or imbalance between responsibility and decision-making authority. Ethical leadership may therefore become constrained not primarily through individual moral failure, but through structural conditions that limit leaders’ ability to act in accordance with their values.

## Discussion

The central finding of this study is that ethical leadership in health and social care was characterised not as an individual trait, but as a systemic, relational and context-dependent phenomenon. Ethics was described as the foundation of leadership, inseparable from legitimate managerial practice, yet difficult to define and enact consistently in everyday organisational contexts. This conceptualisation shifts attention from individual leader characteristics (Brown and Treviño, 2006) towards the broader relational and organisational conditions shaping ethical leadership in practice.

Leaders’ experiences further highlighted the interdependence of these prerequisite conditions, with ethical agency reflecting leaders’ capacity to enact them in practice. From a critical realist perspective, these experiences may reflect underlying organisational and structural mechanisms through which ethical leadership is enabled or constrained. These findings suggest that ethical leadership is shaped by the organisational conditions that enable or constrain leaders’ ability to act ethically in practice. This study advances existing research by offering an empirically grounded conceptualisation of the prerequisites for ethical leadership as an interdependent phenomenon. Unlike prior studies that have focused on identifying individual competencies or organisational barriers in isolation (Barkhordari-Sharifabad *et al.*, 2017; Singh and Vashist, 2024), the findings show how individual capabilities and organisational conditions work together, making ethical leadership contingent on the presence of these conditions.

The findings suggest that ethical leadership permeates organisational levels and daily practices, rather than being solely tied to a leader’s personal morality. This extends prior conceptualisations of ethical leadership, which have predominantly emphasised individual-level attributes such as integrity and fairness (Brown and Treviño, 2006; Eisenbeiss, 2012; Northouse, 2021), by highlighting how ethical practice is also shaped by organisational, including structural, conditions. Rather than functioning solely as an individual-level phenomenon, ethical leadership appeared to emerge through interactions between leaders, relational processes and organisational conditions. Ethical agency can thus be interpreted as the ability to act in accordance with professional and organisational values within complex contexts, where competing demands and limited resources may shape how ethical intentions are enacted in practice.

Trust, respect and human interaction emerged as important prerequisites rather than merely supportive conditions. Ethical leadership appears to be enacted through mutual trust and shared values, that inform daily decision-making, consistent with perspectives emphasising the relational nature of ethical leadership (Hansen and Jørgensen, 2021). Rather than functioning as isolated relational qualities, trust, mutual respect and interaction appeared to shape how organisational conditions were experienced and enacted in everyday leadership practice. In this sense, these relational elements appeared to connect ethical agency with broader organisational contexts, influencing how values are interpreted, negotiated and translated into action. This emphasis on relational processes extends previous research on internal leader capacities such as ethical awareness and moral resilience (Barkhordari-Sharifabad *et al.*, 2017; Makaroff *et al.*, 2014), suggesting that relational conditions are not secondary to individual capabilities but constitute an equally important prerequisite.

Organisational and systemic conditions including ethical culture, transparent communication and managerial support were described as important enablers or barriers (Morin and Talbot, 2023; Paatela *et al.*, 2025; Singh and Vashist, 2024). These conditions appeared closely connected to relational processes, influencing trust and shared understanding. Conversely, one-way communication and hierarchical decision-making were described as constraining ethical agency, a pattern consistent with earlier findings on restricted autonomy (Storaker *et al.*, 2022). These organisational constraints appeared to shape relational processes and, consequently, limit the enactment of ethical leadership in practice (Storaker *et al.*, 2022).

The leaders acknowledged existing professional codes of ethics, organisational value statements and legislative frameworks but noted that explicit guidance for ethical leadership was often lacking. This finding points to a persistent gap between formal ethical frameworks and their enactment in everyday leadership practice. While prior research has shown that ethical frameworks remain ineffective unless actively embedded in organisational practice (Barkhordari-Sharifabad *et al.*, 2018; Luu, 2012), the present findings suggest that formal structures alone may not ensure ethical leadership unless supported by ongoing dialogue and leadership practices that actively model organisational values. This highlights a tension between formalised ethics and lived ethical practice, where ethical codes may create expectations of ethical conduct without necessarily ensuring the conditions required to realise it in practice.

Resource sufficiency was identified as a prerequisite for ethical leadership. Adequate staffing, time and supportive structures were described as enabling ethical leadership, while scarcity was associated with compromises and increased stress (Barkhordari-Sharifabad *et al.*, 2017; Morin and Talbot, 2023). The leaders viewed resource sufficiency not merely as an operational issue but as an ethical responsibility toward staff and service users. Resource conditions may thus shape not only what leaders can do, but also whether ethical leadership can be consistently enacted in practice. This is consistent with prior research linking resource-supported ethical leadership to psychological safety, organisational commitment and care quality (Barkhordari-Sharifabad *et al.*, 2018; El-Gazar and Zoromba, 2021; Lotfi *et al.*, 2018).

This study provides empirically grounded insight into the prerequisites for ethical leadership as a context-dependent and multi-level phenomenon. The findings indicate that ethical leadership is enabled and constrained through the interactions between individual, relational and organisational conditions, alongside leaders’ ability to act within them. From a critical realist perspective, ethical leadership is shaped through the interaction of organisational structures, relational processes and leaders’ capacity to act within them. When these conditions are insufficiently present, ethical leadership may remain aspirational rather than consistently enacted, pointing to a practice-oriented understanding of prerequisites that moves beyond normative advocacy.

Future research should examine how the conditions identified here operate and interact across different organisational contexts, leadership levels and health systems – for example, comparing primary care and hospital settings or contrasting welfare state models with differing degrees of centralisation. Developing and validating instruments for assessing these prerequisites would enable systematic measurement across contexts, building on the conceptualisation advanced in this study. Intervention studies evaluating specific organisational measures such as ethics education programmes, structured peer support or ethics consultation services, would help determine which conditions are most amenable to change and under what circumstances. Multi-level and cross-national studies incorporating staff and service-user perspectives would further enhance generalisability. Beyond exploratory designs, longitudinal approaches would offer important insights into how these conditions develop and shift over time in response to organisational and policy changes.

### Implications for health and social care leadership

Organisations may strengthen the prerequisites for ethical leadership by investing in leaders’ professional development, supportive organisational structures, ethical reflection, interactional practices and facilitated peer support across organisational levels. Supporting ethical leadership requires action across structural, relational and organisational dimensions, including adequate resourcing, opportunities for ethical dialogue and conditions that enable leaders to act in accordance with professional values. In practice, this includes manageable spans of control, sufficient time for leadership tasks and structured support for ethical reflection in everyday work. While resource scarcity, hierarchical decision-making and limited organisational support have been shown to undermine ethical leadership, the present study suggests that these challenges are interdependent and require coordinated responses across organisational levels, rather than isolated interventions. Strengthening the prerequisites for ethical leadership should be viewed as a strategic leadership investment supporting the conditions under which ethical leadership can be sustainably enacted (Morin and Talbot, 2023; Seere *et al.*, 2026). This requires a shift from viewing ethical leadership as individual conduct to understanding it as a shared organisational responsibility.

### Limitations and methodological considerations

The study was conducted in the Finnish health and social care context during a period of significant organisational reform, which may limit transferability to more stable health and social care environments or national contexts. The study also intentionally focused on leaders’ perspectives, as ethical leadership prerequisites were examined from the viewpoint of those responsible for their enactment. However, frontline staff and service users may experience these prerequisites differently, for example, perceiving relational trust or organisational ethical culture in ways that diverge from leaders’ accounts.

Member checking was not conducted, as RTA conceptualises themes as interpretive constructs rather than direct representations of participants’ experiences, making participant validation methodologically inappropriate (Braun and Clarke, 2019, 2021). Interviews were conducted both face-to-face and virtually, which may have influenced interaction. However, the depth and variation of participants’ accounts suggested that both approaches enabled rich reflections on ethical leadership. The credibility of the study was further strengthened through systematic reporting guided by established qualitative criteria (Lincoln and Guba, 1985).

The analysis was conducted by one primary coder, which may be considered a limitation. To address this, reflexivity was maintained through positional reflection, and analytical decisions were discussed with other members of the research team throughout the process. Reporting followed SRQR and COREQ guidelines (O'Brien *et al.*, 2014; Tong *et al.*, 2007). The primary researcher’s professional background as a leader in health and social care informed both the depth of engagement with participants and the interpretive lens applied during analysis. While this insider position may have shaped the directions of inquiry and thematic interpretation, it was systematically managed through reflexive journaling and ongoing discussion with other members of the research team throughout the process. This positionality is therefore understood not solely as a limitation but as an analytical resource that enhanced contextual sensitivity, while acknowledging that its influence on the findings cannot be entirely eliminated.

## Conclusion

This study suggests that the prerequisites for ethical leadership in health and social care form an interdependent, multi-level system in which individual, relational and organisational conditions are closely connected. Depending on how these prerequisites are supported, they may either enable or constrain the enactment of ethical leadership in practice. By providing empirically grounded insight into how these conditions are experienced and negotiated by leaders, this study extends the existing understandings of ethical leadership beyond predominantly conceptual and outcome-focused approaches. The findings suggest that ethical leadership is shaped through interactions between leaders, relationships and organisational conditions rather than reducible to individual attributes alone.

These findings align with international frameworks, including the World Health Organization’s Global Strategic Directions for Nursing and Midwifery 2021–2025 (World Health Organization, 2021), by providing empirical insight into the organisational, relational and systemic prerequisites that support ethical leadership in practice. In line with this framework, the findings underscore the need for sustained investment in leadership development, education and supportive organisational conditions to enable ethical leadership in practice. Without sufficient support for these prerequisites, ethical leadership may remain difficult to sustain systematically, even when individual leaders are committed to ethical practice.

## Supplementary Material

Data supplement 1

## Data Availability

The data supporting this study are not publicly available due to the sensitive nature of the interview material. Anonymised excerpts may be made available upon reasonable request to the corresponding author.

## Institutional affiliations

All work was conducted at the Department of Nursing Science, Faculty of Medicine, University of Turku, Finland, and the Wellbeing Services County of Southwest Finland.

## Supplementary material

The supplementary material for this article can be found online.
