# Supplementary material for: Prerequisites for ethical leadership: insights from health and social care leaders
Source: Leadersh Health Serv (Bradf Engl). 2026 Jun 30;35(9):81–98. doi: 10.1108/LHS-12-2025-0201 (PMC13308684; doi:10.1108/LHS-12-2025-0201)
Supplement: Data supplement 1 [file lhs-12-2025-0201_supplementary_file_1.docx]

Supplementary File 1. Reporting standards: SRQR and COREQ

Standards for Reporting Qualitative Research (SRQR; O’Brien et al., 2014)

| SRQR item | Description | Addressed in manuscript |
| --- | --- | --- |
| 1. Title and Abstract | Title and abstract clearly indicate qualitative approach and study focus | Title and abstract describe the qualitative approach and aim of exploring leaders’ perceptions of ethical leadership prerequisites. |
| 2. Problem Formulation | Description of the significance of the problem and existing literature. | Introduction, pp. 2–3 |
| 3. Purpose or Research Questions | Clear statement of study purpose and research questions | End of Introduction, p. 3 |
| 4. Qualitative Approach and Research Paradigm | Specification of the approach and paradigm | Methods, “Design and approach”: reflexive thematic analysis within a critical realist framework. |
| 5. Researcher Characteristics and Reflexivity | Information about researchers’ backgrounds, positions, and reflexivity | Methods, “Researcher position and reflexivity”, Discussion, Limitations |
| 6. Context | Description of setting and relevant contextual features | Methods, “Study Setting and Recruitment” |
| 7. Sampling Strategy | Explanation of sampling method, inclusion logic, and sample size rationale | Methods, “Study setting and recruitmen” |
| 8. Ethical Issues | Ethical approval and informed consent process | Methods, “Ethical statement” |
| 9. Data Collection Methods | Description of data collection, instruments, and procedures | Methods, “Data collection” |
| 10. Data Collection Instruments and Technologies | Description of interview guide and tools used | Methods, “Data collection” |
| 11. Units of Study | Number and characteristics of participants | Methods, “Participants” |
| 12. Data Processing | Procedures for data transcription, management, and storage | Methods, “Data analysis” |
| 13. Data Analysis | Analytic process and framework clearly described | Methods, “Data analysis”: Braun & Clarke’s six-phase reflexive thematic analysis. |
| 14. Techniques to Enhance Trustworthiness | Use of strategies such as reflexivity, transparency, and audit trail | Methods, “Trustworthiness” |
| 15. Synthesis and Interpretation | Description of the main themes and interpretation | Results and Discussion sections |
| 16. Links to Empirical Data | Illustrative quotations supporting themes | Results section: representative quotes provided |
| 17. Integration with Prior Work | Interpretation related to existing theory and literature | Discussion, pp. 10–12 |
| 18. Limitations | Discussion of study limitations | Discussion, “Limitations” |
| 19. Transferability | Discussion of applicability to other contexts | Discussion, “Implications” |
| 20. Conflicts of Interest | Disclosure of any potential conflicts | Ethics statement, final section |
| 21. Funding | Sources of support and funding acknowledged | Acknowledgements section |

COREQ Checklist, 32 items (Tong et al. 2007)

| Item no.. | Checklist item | Response |
| --- | --- | --- |
| Domain 1: Research team and reflexivity |  |  |
| 1 | Which author/s conducted the interview or focus group? | The first author conducted all interviews. |
| 2 | What were the researcher’s credentials? | Master’s-level training; doctoral studies ongoing. |
| 3 | What was their occupation at the time of the study? | Researcher with experience in health and social care leadership. |
| 4 | Was the researcher male or female? | Female. |
| 5 | What experience or training did the researcher have? | Formal education in health and social care leadership; over 15 years of leadership experience in the sector; completed advanced training in qualitative research methods and reflexive thematic analysis. |
| Domain 2: Study design |  |  |
| 6 | What methodological orientation was stated to underpin the study? | Reflexive Thematic Analysis (Braun & Clarke) within a critical realist framework. |
| 7 | How were participants selected? | Purposive sampling. |
| 8 | How were participants approached? | Email and phone contact via organisational gatekeepers. |
| 9 | How many participants were in the study? | 16 participants. |
| 10 | How many people refused to participate or dropped out? Reasons? | None refused or dropped out after agreeing to participate. One invited participant did not respond to the contact request. |
| 11 | Where was the data collected? | In person (at participants’ workplaces or meeting rooms) or via encrypted videoconferencing. |
| 12 | Was anyone else present besides the participants and researchers? | No. |
| 13 | What are the important characteristics of the sample? | Frontline and middle-management leaders in health and social care across multiple regional organisations; see Table 4 for details. |
| 14 | Were questions, prompts, guides provided by the authors? Was it pilot tested? | Yes. Preliminary test interviews were conducted with health and social care leaders who did not participate in the study; no revisions were required. The first four study interviews were additionally monitored closely for consistency with the guide, further confirming its adequacy. |
| 15 | Were repeat interviews carried out? If yes, how many? | No repeat interviews. |
| 16 | Did the research use audio or visual recording to collect the data? | Audio recording was used with participant consent. |
| 17 | Were field notes made during and/or after the interview or focus group? | Yes, reflexive notes and memos were maintained during and after interviews. |
| 18 | What was the duration of the interviews or focus group? | 45–75 minutes. (mean = 55) |
| 19 | Was data saturation discussed? | Sampling was guided by the principle of information power (Malterud et al., 2016); sufficient variation and richness were achieved to address the research questions. |
| 20 | Were transcripts returned to participants for comment and/or correction? | No. Transcripts were anonymised and not returned for comment. |
| Domain 3: Analysis and findings |  |  |
| 21 | How many data coders coded the data? | One primary coder (first author). |
| 22 | Did authors provide a description of the coding tree? | Yes. The process of generating codes, grouping into themes, and final theme structure is described in detail in the Methods section, in Tables 2 and 3. |
| 23 | Were themes identified in advance or derived from the data? | Themes were derived inductively from the data. |
| 24 | What software, if applicable, was used to manage the data? | NVivo 14. |
| 25 | Did participants provide feedback on the findings? | No formal member checking of findings was conducted. |
| 26 | Were participant quotations presented to illustrate the themes/findings? | Yes. Anonymised quotes are presented in Results (Table 3 and throughout the Results text). |
| 27 | Was there consistency between the data presented and the findings? | Yes. Themes and interpretations were supported with extensive participant quotations and reflexive analysis. |
| 28 | Were major themes clearly presented in the findings? | Yes. Five clearly defined themes with subthemes are presented. |
| 29 | Is there a description of diverse cases or discussion of minor themes? | Yes. Differences in experience and context are discussed in subthemes and quotes. |
| 30 | Was ethics approval obtained and from which institution? | Ethical review was obtained; the approving institution is removed for blinded review. Organisational approvals were secured. |
| 31 | Was informed consent obtained from all participants? | Yes. Written informed consent was obtained from all participants. |
| 32 | How was participant confidentiality protected? | Audio recordings were stored securely and pseudonymised immediately. Transcripts removed all personal identifiers. Data were handled in compliance with GDPR and securely stored on encrypted platforms. Participants’ quotes were anonymised in reporting. |

Supplementary File 2. Quality Checklist for RTA (Braun & Clarke, 2021)

| Checklist question | Assessment (researcher’s self-assessment) |
| --- | --- |
| 1. Is the research question clear, and is thematic analysis appropriate? | Yes, the research aim is clearly defined, and the analytic method supports them. |
| 2. Has reflexive thematic analysis been used consistently in the analysis? | Yes, reflexive thematic analysis is named and applied consistently. |
| 3. Is the use of reflexive thematic analysis justified in relation to the theoretical framework? | Yes, the method fits a critical realist framework with constructionist orientation. |
| 4. Is there a clear, well-reasoned rationale for choosing the analytic method? | Yes, the rationale is presented from an epistemological stance and appropriate for the topic’s complexity. |
| 5. Are the theoretical assumptions underpinning thematic analysis described clearly? | Yes, the analysis is grounded in critical realist framework with constructionist, interpretive, and reflexive perspectives. |
| 6. Has the researcher’s positionality and role been identified and addressed? | Yes, the researcher’s background and positionality are identified and reflected on as part of the analysis. |
| 7. Is the analytic method described in sufficient detail to allow quality appraisal? | Yes, the method and stages of the analysis are described in detail. |
| 8. Is the progression of the analysis described transparently, step by step? | Yes, each analytic stage is reported transparently and systematically. |
| 9. Is the analysis data-driven, theory-informed, or both—and is this aligned with the study? | Yes, the analysis coherently combines data-driven and theory-driven approaches. |
| 10. Is the coding and theme-development process described clearly? | Yes, coding and theme construction are documented in NVivo and in a research diary. |
| 11. Were themes produced through active interpretation rather than simply “emerging” from the data? | Yes, themes were constructed interpretively; they are not described as “emerging” from the data. |
| 12. Are the themes conceptually coherent and distinguishable from one another? | Yes, the final themes are conceptually coherent and distinct. |
| 13. Is each theme supported by sufficient and illustrative data extracts/quotes? | Yes, multiple representative and vividly presented participant quotes support the themes. |
| 14. Does the analysis include a persuasive interpretive narrative in addition to quotations? | Yes, the analysis includes interpretive narrative rather than mere description. |
| 15. Are the themes situated within a broader narrative or conceptual framework? | Yes, the themes are situated within a system-level framework and the structure of the analysis. |
| 16. Is the researcher’s interpretation distinguishable from the data (e.g., participant quotes)? | Yes, the researcher’s interpretation is clearly distinguishable from the participants’ quotes. |
| 17. Is reflexive practice visible throughout the analytic process? | Yes, reflexivity is a key feature of the analysis and is evident throughout. |
| 18. Is there evidence of iterativity and in-depth interpretation? | Yes, the analysis progressed through multiple rounds, which deepened iteratively. |
| 19. Does the analysis address the research questions comprehensively? | Yes, the themes address the research questions comprehensively and in depth. |
| 20. Has analytic quality been ensured through appropriate and documented means? | Yes, quality was ensured through a research diary, visual models, and the COREQ and SRQR guidelines. |

Supplementary File 3. Detailed coding examples and illustrative quotes (Source: Authors' own work)

| Example Participant Quote | Analytical Interpretation | Code | Subtheme | Theme |
| --- | --- | --- | --- | --- |
| *"And when it affects us, it quickly spreads to our subordinates. That uncertainty spreads and is felt in the staff... It does affect clients—after all, we’re here for the clients."* (Participant 6) | Used when participants describe how uncertainty or lack of ethical principles spreads in the organisation, cascading from leaders to staff and ultimately to clients. Highlights systemic ethical risks. | Chain of ethical problems and uncertainty in the organisation | Organisational-level impact and visibility of ethical leadership | Theme 1. The fundamental nature of ethical leadership and the difficulty of defining it in health and social care |
| *"It’s equal to good leadership."* (Participant 10) | Applied when ethical leadership is framed as an inseparable aspect of good leadership overall, not a separate style. | Ethical leadership equals good Leadership | Ethics as a guiding principle and core orientation of leadership |  |
| *"Always a bit like, from whose perspective is it ethical and whose values are these."* (Participant 13) | Highlights recognition that values are context-dependent and multifaceted; ethics is negotiated rather than fixed. | Contextual interpretation of values | Complexity and boundary work in ethical leadership |  |
| *"It requires self-examination and self-awareness to think about these things—it doesn’t just come as a birthright."* (Participant 16) *"Every training, every lesson adds to the previous ones. It’s not just more knowledge, but more understanding of how much there is to learn."* (Participant 14) | Used when participants link ethical leadership to active development, reflection, or learning. | Continuous learning as part of ethical leadership | Developing ethical leadership competence | Theme 2. The leader’s internal ethical capability |
| *"In my view, a good, responsible ethical leader also recognises their own limits."* (Participant 15) | Applied when participants discuss planning, setting boundaries, or managing their own work ethically. | Self-management as part of ethical leadership | Resilience, personal ethics, and recognising limits |  |
| *"The whole organisation is in the same boat together; you can't be ethical alone in an unethical group."* (Participant 14) | Used when participants describe support, connection, or being alongside others to face ethical challenges. | Supporting a sense of belonging in ethical leadership | Community, peer support, and psychological safety | Theme 3. Human interaction and trust as the basis of ethical leadership |
| *"I think there has to be that mutual trust to lead ethically. Collaboration and fairness."* (Participant 13) | Applied when participants highlight experiences or hope for respectful, equal treatment regardless of role or background. | Mutual respect and equal treatment | Trust and respect as cornerstones of ethical leadership |  |
| *"Maybe ethical leadership isn’t explicitly talked about as a value there, but it’s probably there in the background anyway."* (Participant 11) | Used when ethics is described as integrated into structures, guidance, and decision-making—not just individual choice. | Structural embeddedness of ethics | Structurally embedded ethical culture | Theme 4. Organisational structures and culture as the context of ethical leadership |
| *"We’re in a pretty severe financial squeeze right now, so that’s really the main thing steering decisions."* (Participant 12) | Applied when participants describe how structures or budget constraints limit ethically sustainable choices. | Structural and financial limitations | Economic and political constraints shaping leadership |  |
| *"In the name of ethics, you have to look at how the staff is doing so that things like compassion fatigue don’t happen."* (Participant 11) | Applied when leaders describe personal responsibility for staff wellbeing, resilience, and ethical practices. | Ethical responsibility for staff | Responsibility for staff and the ethical climate | Theme 5. The leader’s ethical agency in a hierarchical system |
| *"There’s so much responsibility, so there has to be power, too. That’s something that’s been off lately—tons of responsibility, but less authority."* (Participant 5) | Used when participants describe lacking decision-making power relative to their responsibilities. | Limited authority in leadership | Hierarchical and communicative constraints |  |
| *"If only you had a table that told you: if this is the problem, do this—but it doesn’t work that way. Situations aren’t black and white."* (Participant 4) | Used when participants describe difficulty choosing or applying leadership styles in ethically complex situations. | Challenges of adapting leadership styles | Applying leadership theories in practice |  |
